# Tumor cell-derived EMP1 is essential for cancer-associated fibroblast infiltration in tumor microenvironment of triple-negative breast cancer

**DOI:** 10.1038/s41419-025-07464-9

**Published:** 2025-02-27

**Authors:** Qi Wang, Dandan Li, Haixiu Ma, Zengyan Li, Juan Wu, Jinwan Qiao, Jun Liu, Jing Zhao, Ronghua Ma, Lin Tian, Lei Zhang, Jianye Yang, Jianing Wang, Shanshan Qin, Zhanhai Su

**Affiliations:** 1 Research Center for High-Altitude Medicine, Key Laboratory of High-Altitude Medicine, Ministry of Education, Laboratory for High Altitude Medicine of Qinghai Province, Key Laboratory of Application and Foundation for High Altitude Medicine Research in Qinghai Province (Qinghai-Utah Joint Research Key Lab for High-Altitude Medicine), Qinghai University, Xining, China; 2 Department of Pathology, Renmin Hospital, Hubei University of Medicine, Shiyan, Hubei China; 3 Shiyan Key Laboratory of Comprehensive Prevention and Treatment of Oral Cancer, Department of Stomatology, Taihe Hospital, Hubei University of Medicine, Shiyan, Hubei China; 4 Experimental Animal Center & Institute of Clinical Medicine, Renmin Hospital, Hubei University of Medicine, Shiyan, Hubei China

**Keywords:** Breast cancer, Cancer microenvironment, Interleukins

## Abstract

The role of epithelial membrane protein 1 (EMP1) in tumor microenvironment (TME) remodeling has not yet been elucidated. In addition, the biological function of EMP1 in triple-negative breast cancer (TNBC) is largely unclear. In this study, we examined the infiltration landscape of cell types in the TME of breast cancer, and found that EMP1 expression was positively correlated with stromal and microenvironmental scores. Infiltration analysis and immunohistochemical (IHC) staining of serial sections confirmed the critical role of EMP1 in cancer-associated fibroblast (CAF) infiltration. Cell co-culture assays, xenograft tumor experiments, loss-of-function, gain-of-function, RNA sequencing studies, and rescue assays were performed to confirm the role of EMP1 in CAF infiltration in vitro and in vivo. These findings revealed that EMP1 depletion in TNBC cells resulted in considerable inhibition of CAF infiltration in vivo and in vitro. Mechanistically, EMP1 knockdown induced a substantial decrease in IL6 secretion from TNBC through the NF-κB signaling pathway, hindering CAF proliferation and subsequently inhibiting TNBC progression and metastasis. These cumulative results indicate that EMP1 functions as an oncogene in TNBC by mediating the cell communication of TNBC and CAFs. Targeted inhibition of EMP1 by suppressing CAF infiltration is a promising strategy for TNBC treatment.

## Highlights

- We present a comprehensive infiltration landscape in TME cell types in TNBC, and highlighted the correlation between EMP1 expression and TME cell infiltration.
- EMP1 expression is closely associated with CAF infiltration in BC, particularly TNBC. EMP1 in TNBC is essential for CAF infiltration both in vivo and in vitro.
- EMP1 promotes TNBC cell proliferation, migration and invasion by regulating NF-kB signaling.
- EMP1 mediates the cell communication between TNBC cells and CAFs by enhancing cancer cell-derived IL6 secretion. Our findings suggested that blockade of EMP1 may represent a promising therapeutic strategy for overcoming TNBC.

## Introduction

Breast cancer (BC) is one of the most prevalent tumors in women and is primarily divided into invasive and non-invasive types [1, 2]. Invasive BC is the most common type of BC and originates in the breast epithelium or duct epithelium [3, 4]. Based on molecular typing, invasive BC can be divided into four types: luminal A (lumA), luminal B (lumB), HER2-positive, and TNBC [5]. The estrogen receptor (ER), progesterone receptor (PR), and epidermal growth factor receptor (HER2) in TNBC are negative and predominantly found in younger women [6]. Compared to other subtypes of BC, the risk of distant metastasis and death in TNBC is higher [7]. According to the PAM50 molecular typing standard, breast cancer can be divided into five subtypes, including luminal A, luminal B, HER2-positive, basal like, and normal like [8]. Most patients with TNBC have a basal-cell-like PAM50 subtype [9]. Owing to the absence of specific molecular targets (ER, PR, and HER2), TNBC is unresponsive to endocrine therapy or relevant targeted therapy [10–12]. Therefore, it is very important to explore the potential therapeutic targets in patients with TNBC.

The tumor microenvironment (TME) refers to the surrounding microenvironment in which tumor cells exist, including surrounding blood vessels, various immune cells (such as T cells, B cells, and macrophages), fibroblasts, various signaling molecules, and the extracellular matrix [13–18]. The TME is closely associated with tumorigenesis and metastasis [19–21]. Tumor cells can alter and maintain their own survival and development conditions through autocrine and paracrine processes [22–24]. Cancer-associated fibroblasts (CAFs) are the main stromal cells in the tumor microenvironment (TME), and play an important role in supporting tumor growth, reshaping the extracellular matrix, creating metastatic plaques, and protecting tumor cells from the immune system [25–27]. Therefore, targeting CAFs is considered an effective strategy for treating patients with TNBC [28–30].

EMP1 (epithelial membrane protein 1) belonged to the transmembrane superfamily, which comprises four major families: PMP22/GAS3, claudins, connexins, and tetraspanins. PMP22 (peripheral myelin protein 22) is also known as GAS3 (growth arrest-specific gene 3), and the GAS3/PMP22 family comprises seven members, including PMP22, EMP1 (TMP), EMP2 (XMP), EMP3 (YMP), PERP, BCMP1, and MP20 [31]. Members of the GAS3/PMP22 family are closely associated with the development of various diseases, including hereditary peripheral nervous system disorders and other disorders related to cell growth, differentiation, and apoptosis [31, 32]. Several studies have shown that the abnormal EMP expression plays an essential role in tumor progression and metastasis [33–35]. However, the biological role and molecular mechanism of EMP1 in TNBC have not yet been reported. In this study, we investigated the biological function and molecular mechanism of EMP1 in the cell-cell communication between TNBC cells and CAFs. We have identified EMP1 as an oncoprotein and potential therapeutic target in patients with TNBC. Our findings highlight that TNBC cell-expressed EMP1 is required for CAF proliferation by enhancing IL6 secretion through activating the NF-κB signaling axis.

## Methods

### Survival analysis

For each subtype, BC patients in the TCGA_BRCA group were divided into two groups (low_EMP1 group and high_EMP1 group) according to the normalized expression level (TPM value) of EMP1. All the possible cut-off values between the low_EMP1 group and high_EMP1 group were computed using the survive R package, and the best-performing threshold was selected as the final cut-off value. The *P* values and HR values of the survival analysis were evaluated using the Log-rank test.

### Patients

The TCGA breast cancer (TCGA_BRCA) cohort, which includes gene expression profiles and prognostic information from 1044 BC samples with detailed PAM50 expression, was used for survival analysis. A series of previously applied serum specimens and FFPE tissue microarrays (TMA) from the tissue bank of the Department of Pathology, Renmin Hospital, Hubei University of Medicine, which included 74 BC patients (Lumina A (*N* = 14), Lumina B (*N* = 16), HER2+ (*N* = 15), and TNBC (*N* = 29)) who underwent tumor resection during 2018–2023, were used for immunochemistry (IHC) analysis of EMP1 and αSMA expression. The patient follow-ups were conducted every three months during the first postoperative year and one year thereafter until May 30, 2023. This study was approved by the Research Ethics Committee of the RHHUM (SYRRMYY2022-042), and all patients provided signed informed consent. Detailed clinical information for BC patients in this study is presented in Table S1.

### Immunohistochemistry staining and evaluation

Primary EMP1 antibodies (dilution 1:200, Cat# ab230445, Abcam, Cambridge, UK) and α-SMA antibodies (1:400, Cat# 27095-1-AP, Proteintech, Wuhan, China) were used to evaluate the EMP1 and α-SMA protein level in TNBC tissues. The protein expression levels of EMP1 or α-SMA were quantified using the product of the staining intensity score and positive cell percentage score. Scores for staining intensity: 0 = no staining, 1 = weak staining, 2 = medium staining, and 3 = strong staining. For the percentage of positive cells, score 0 = 0–5% positive cells, 1 = 6–25% positive cells, 2 = 26–50% positive cells, 3 = 51–75% positive cells, and 4 = 76–100% positive cells.

### Cell culture and isolation of CAFs

The Human TNBC cell lines of MDA-MB-231 (CL-0150, Pricella, Wuhan, China), MDA-MB-468 (CL-0290, Pricella, Wuhan, China), and MDA-MB-453 (STCC10510, ZibinBio, Wuhan, China), were cultured in DMEM medium (Cat# G4515, Serveicebio, Wuhan, China), as previously reported [36, 37]. Cancer-associated fibroblasts (CAFs) were isolated from human TNBC patients who underwent surgery at the Department of Mammary Surgery, RHHUM. Tissues were initially cleaned in phosphate buffer saline (PBS, Cat# G4202, Serveicebio, Wuhan, China), supplemented with 100 U/mL penicillin and 100 µg/mL streptomycin (Cat# G4003, Serveicebio, Wuhan, China) and then cut into almost 1 mm³ small pieces. Type II and IV collagenase (0.2%, Cat# 9001-12-1, MCE, Shanghai, China) and hyaluronidase (100 U/ml, Cat# 37326-33-3, Baomanbio, Shanghai, China) were used to digest tissues at 37 °C in a water shaker for 2 h. Subsequently, 10% fetal bovine serum (Cat#164210, Pricella, Wuhan, China) was added to the erythrocyte digestion process. After filtration through a 100-mesh sterile mesh filter and centrifugation (1200 × rpm, 10 min), cells were transferred to a new culture dish. CAFs were resuspended in DMEM/F12 medium (Cat# PM150310, Pricella, Wuhan, China) and purified after two generations, CAFs were identified through the expression of CAFs markers, such as FAP, α-SMA, Vimentin, and FSP-1, which clearly distinguish them from normal fibroblasts and then used for functional experiments [38, 39].

### CAF-cancer cell co-culture system

To investigate the biological effect of TNBC cells on CAFs, a transwell chamber with a membrane comprising 0.8 μm pore size holes (NEST, Wuxi, China) was used to construct the non-contact co-culture system. The CAFs were introduced into the lower chamber of the transwell plate at a density of 2 × 10^5^/mL, while the TNBC cells were placed into the upper chamber of the transwell plate at a density of 1 × 10^5^/mL. This allowed the exchange of supernatants but not cells, and after one week of co-culture, the cells were collected for subsequent experiments.

### Double immunofluorescence staining for tissues

Paraffin-embedded cancer tissue samples were deparaffinized and rehydrated for antigen recovery. The tissues were then incubated with 3% BSA for 30 min at room temperature to block non-specific binding. After the blocking step, the slides were incubated with the first primary antibody-goat anti-human EMP1 (1:200, Cat# ab230445, Abcam, Cambridge, UK) - at 4 °C overnight. After treatment with CY3-TSA solution and microwave heating, the primary and secondary antibodies and tissues were removed. Subsequently, the slides were incubated with a second primary antibody αSMA (1:400, Cat# 27095-1-AP, Proteintech, Wuhan, China) overnight at 4 °C, and then with a second corresponding HRP-conjugated secondary antibody. The samples were treated with FITC-TSA solution followed by one round of microwave heating. Finally, the slides were incubated with DAPI solution for 10 min at room temperature. Images were captured using a light microscope (Olympus BX640) and a slide scanner (Zeiss AxioScan).

### Antibodies and reagents

Recombinant protein IL6 (10 ng/mL, Cat# HY-P7044) and recombinant protein TNFα (10 ng/mL, Cat# HY-P7058) proteins were purchased from MedChemExpress (MCE, New Jersey, USA). Western blotting antibodies against p-AKT ser473 (Cat# 4060, CST), AKT (Cat# 4685, CST) were purchased from Cell Signaling Technology (CST, Boston, MA, USA). The additional antibodies used in this study included EMP1 (Cat# sc-13133, Santa Cruz, Dallas, TX, USA), NFκB (P65) (Cat# sc-7304, Santa Cruz, Dallas, TX, USA), p-IκBα and total IκBα (Cat# sc-8007, Santa Cruz, Dallas, TX, USA), IL6 (Cat# sc- 56196, Santa Cruz, Dallas, TX, USA), FSP-1 (Cat# T40044, Abmart, Shanghai, China), monoclonal mouse anti-β-actin (1:5000 dilution, Cat# AF7018, Affinity, Liyang, China), and secondary antibody-goat anti-Rabbit & Mouse (Cat# M21003, Abmart, Shanghai, China).

### RNA Sequencing

Total RNA from MDA-MB-231 TNBC cell line was extracted using TRIzol reagent (Servicebio, Wuhan, China) in accordance with the previously reported manufacturer’s instructions [40–42]. After quality control evaluation, the RNA samples were sent to a biotech company (Biomarker Technologies, Beijing, China) for transcriptome sequencing. After ribosomal RNAs were removed, the prepared RNA-seq libraries were sequenced using an Illumina Novaseq 6000 sequencer. Detailed steps for library construction and sequencing were performed as previously reported [43–45]. The raw data were filtered using Fastp software. Differences in gene pathways were assessed by Kyoto Encyclopedia of Genes and Genomes (KEGG) analysis [46]. *P* values < 0.05 and fold changes ≥2 were deemed as screening criteria for the examination of gene expression. The original RNA-seq data for global gene expression profiling are presented in Table S2.

### In vivo experiments

All animal experiments were approved by the Ethics Committee of the RHHUM Laboratory Animal Center, Shiyan, China (No. SYRRMYY2022-042) and were conducted in compliance with the institutional guidelines. A total of 5 × 10^6^ MDA-MB-231 cells, either alone or mixed with (1 × 10^6^) CAFs cells (5:1 ratio), were suspended in PBS and injected into the fifth mammary fat pads of 4–6-week-old BALB/c nude mice for orthotopic xenotransplantation (n = 6/group). Additionally, to evaluate the role of the EMP1 gene in tumor invasion, (1 × 10^6^) shRNA-transfected MDA-MB-231 cells (without CAFs) were injected into the tail vein for lung colonization studies. Body weight and tumor size were monitored every three days from the start of the experiment. Tumor length (L) and width (W) were measured, and tumor volume was calculated using the formula (0.52 × *L* × *W*^2^)

### Enzyme-linked immunosorbent assay (ELISA)

In vitro knockdown of the EMP1 gene was performed in the MDA-MB-231 and MDA-MB-468 cell lines. When the cells reached 90% confluence, the medium was replaced with serum-free DMEM, and the cells were cultured for 24 h to eliminate the effect of serum on cytokine secretion. After 24 h, the culture supernatant was collected, centrifuged at 1000 × *g* for 10 min at 4 °C, and transferred to sterile test tubes. The samples were immediately stored at −80 °C for subsequent enzyme-linked immunosorbent assay (ELISA) analysis. Serum IL6 levels were quantified using an ELISA kit (Cat# KTE6017, Abbkine, Wuhan, China) following the manufacturer’s protocol. Human plasma samples (BC patients, *n* = 16; healthy donors, *n* = 10) and plasma from tumor-bearing nude mice (EMP1-knockout and non-EMP1-knockout, *n* = 6) were added to the plate wells and incubated to allow IL6 binding. Biotinylated detection antibodies were then added to form immune complexes with the bound IL6. Streptavidin was subsequently introduced, leading to a colorimetric reaction with horseradish peroxidase (HRP) and tetramethylbenzidine (TMB) substrate solutions, which were measured using a microplate reader at 450 nm. Similarly, TNF-α levels were measured using a TNF-α ELISA kit (Cat# KTE6032, Abbkine, Wuhan, China) with the same procedure to maintain methodological consistency. All experiments were performed in triplicate, and each sample was analyzed in triplicate wells to minimize experimental variability. Protein concentrations were calculated using a standard curve.

### Statistical analysis

All experiments were repeated at least three times and GraphPad Prism (version 10.0) statistical software was used to perform all statistical analyses. Data are shown as the means ± standard error of the mean (SEM). The paired t test, Mann–Whitney test, two-tailed chi-square test, and unpaired Student’s *t*-tests were used for two-group comparisons, as appropriate. One-way ANOVA analysis followed by Dunnett’s multiple comparisons test was used to compare three or more groups. In all cases, *P* values less than 0.05 were considered statistically significant. (The other methods in this study can be found in the Supplementary Materials and Methods document.)

## Results

### EMP1 closely associates with CAF infiltration and poor survival in BC patients

**Fig. 1:**
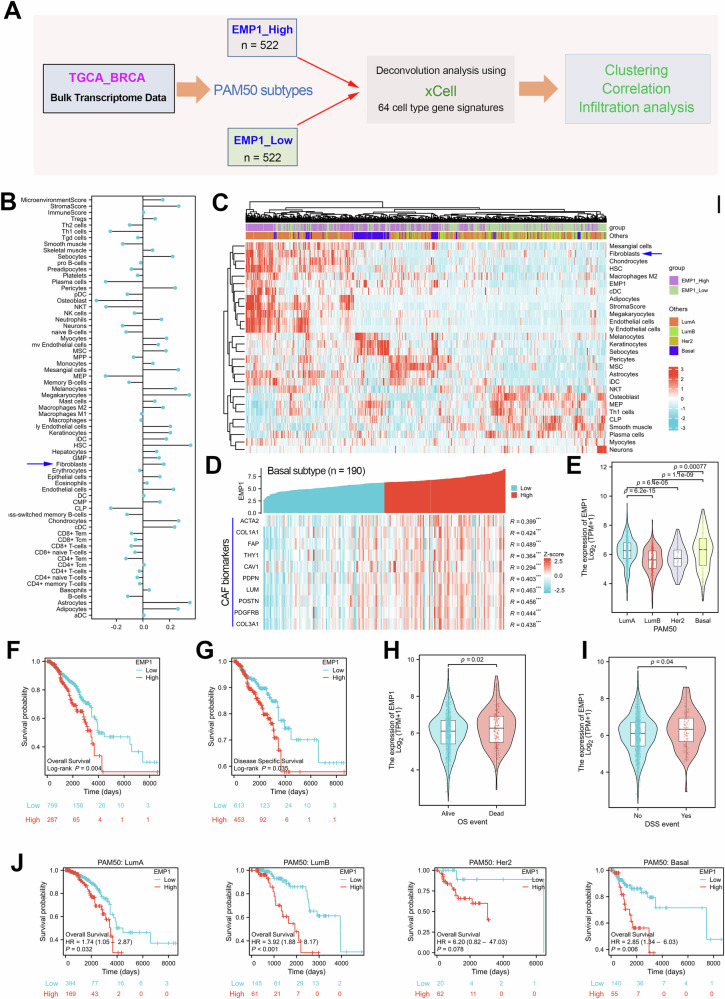
The TME infiltration landscape, expression signature, and prognosis of EMP1 in breast cancer. **A** Schematic diagram of the correlation analysis between EMP1 expression and different tumor microenvironments (TMEs) with regard to cell infiltration. **B** Correlation between EMP1 expression and the infiltration of different TME-related cell types. **C** The TME cell-infiltrating landscape in BC patients with high or low EMP1 expression. **D** Gene expression correlation analysis between EMP1 and the classic CAF biomarkers in the TCGA_BRCA cohort. **E** Expression analysis of EMP1 in different PAM50 subtypes of BC. **F**, **G** BC patients with relatively high EMP1 expression possessed shorter overall survival and disease-free survival times. **H**, **I** Overall survival and disease-free survival of patients with BC. Higher EMP1 expression in patients with BC did not correlate with overall or disease-free survival. **J** Survival analysis of EMP1 for each PAM50 subtype in patients with BC.

According to the clinical information of the BC patients in the TCGA cohort, a total of 1044 patients contained clear PAM50 subtype information. We divided these patients into two groups (EMP1_high group and EMP1_low group) based on the expression level of EMP1, and performed deconvolution analysis was performed using the xCell algorithm based on the bulk RNA-seq data in the TCGA dataset (Fig. 1A). The xCell algorithm is a novel gene signature-based method used to evaluate the infiltration levels of 64 immune and stromal cell types [47]. Next, we analyzed the correlation between the expression level of EMP1 and the infiltration level of each cell type in the TCGA_BRCA cohort. The results showed that EMP1 expression was positively correlated with the tumor microenvironment score, stroma score, and the infiltration of macrophages, endothelial cells, and fibroblasts (Fig. 1B). The infiltration of the corresponding cell types that is significantly correlated with EMP1 expression is shown in the heatmap (Fig. 1C).

As shown in Fig. 1B, C, the expression level of EMP1 was significantly positively correlated with the fibroblast infiltration level, tumor microenvironment score, and stromal cell score. Consistently, gene expression correlation analysis showed that EMP1 was highly co-expressed with the fibroblast biomarkers, such as ACTA2 (αSMA, Fig. 1D). Expression analysis indicated that EMP1 was highly expressed in Basal-like patients (Fig. 1E). Survival analysis confirmed that BC patients with relatively high EMP1 expression possessed a shorter overall survival and disease-free survival time (Fig. 1F–I). Moreover, in the each PAM50 subtype, BC patients with high EMP1 expression predicted a poor prognosis (Fig. 1J). These results together indicated that EMP1 was correlated with CAF infiltration and predicted a poor prognosis in BC. These results implied that EMP1 may play a role in regulating the tumor microenvironment in BRCA.

### EMP1 was highly expressed and positively correlated with CAF infiltration in TNBC patients

**Fig. 2:**
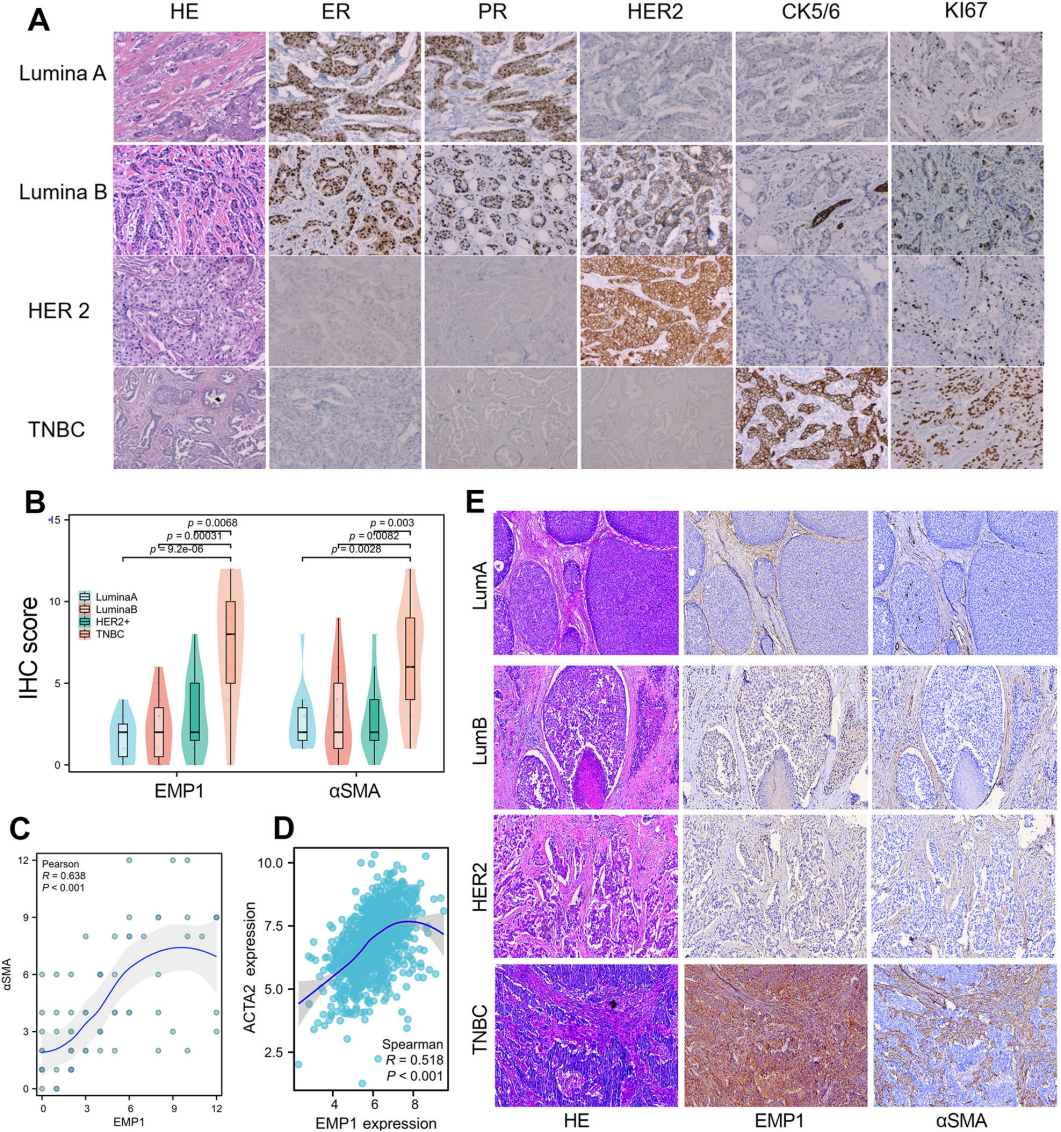
EMP1 was highly expressed and correlated with CAF infiltration in TNBC. **A** Identification and classification of different subtypes of BC (*N* = 74) based on the immunohistochemical results using PR, ER, HER2, Ki-67, and CK5/6 antibodies. A total of 14 patients with lumina A type, 16 with of lumina B type, 15 with HER2+ type, and 24 with TNBC type were identified in our BC cohort. **B** EMP1 and αSMA protein levels were evaluated in the BC cohort using IHC staining. Expression analysis of EMP1 and αSMA in each subtype of BC was conducted. **C** The protein levels of EMP1 were significantly positively correlated with the protein levels of αSMA in BC (*N* = 74). **D** EMP1 mRNA expression was highly co-expressed with ACTA2 mRNA expression in the TCGA_BRCA cohort. **E** Observation of the expression patterns of EMP1 and αSMA in different subtypes of BC using serial pathological sections. ****P < 0.0001; ***P < 0.001; **P < 0.01; *P < 0.05.

In order to further validate the expression characteristics of EMP1 in BC, we conducted immunohistochemical experiments using EMP1 antibodies in our own BC cohort. According to the expression signatures of PR, HER2, ER, CK5/6, and KI-67, this BC cohort (*N* = 74) was divided into four subtypes, including Lumina A (*n* = 14), Lumina B (*n* = 16), HER2-positive (*n* = 15), and TNBC (*n* = 29) patients (Fig. 2A). Subsequently, we examined the expression patterns of EMP1 and αSMA proteins (a classic biomarker for CAF cell type) in each subtype of BC cohort. The results showed that the protein level of EMP1 and αSMA in the TNBC patients were higher than those in the other three subtypes of BC patients (Fig. 2B). Furthermore, expression correlation analysis confirmed that the level of EMP1 protein was highly positively correlated with the level of αSMA protein (Fig. 2C). Similarly, the expression analysis based on bulk RNA-seq data also showed a significant positive correlation between EMP1 mRNA levels and ACTA2 (αSMA) expression levels (Fig. 2D). These results together showed that EMP1 expression was positively correlated with CAF infiltration in BC. To verify this finding, we performed continuous sectioning of BC tissues of different subtypes and immunohistochemical staining based on the EMP1 and αSMA antibodies, respectively. Consistently, the results showed that there was a high co-expression and co-localization of EMP1 protein and SMA protein in BC, especially in the TNBC tissues (Fig. 2E).

### EMP1 positively regulates TNBC cell proliferation, migration, and invasion

**Fig. 3:**
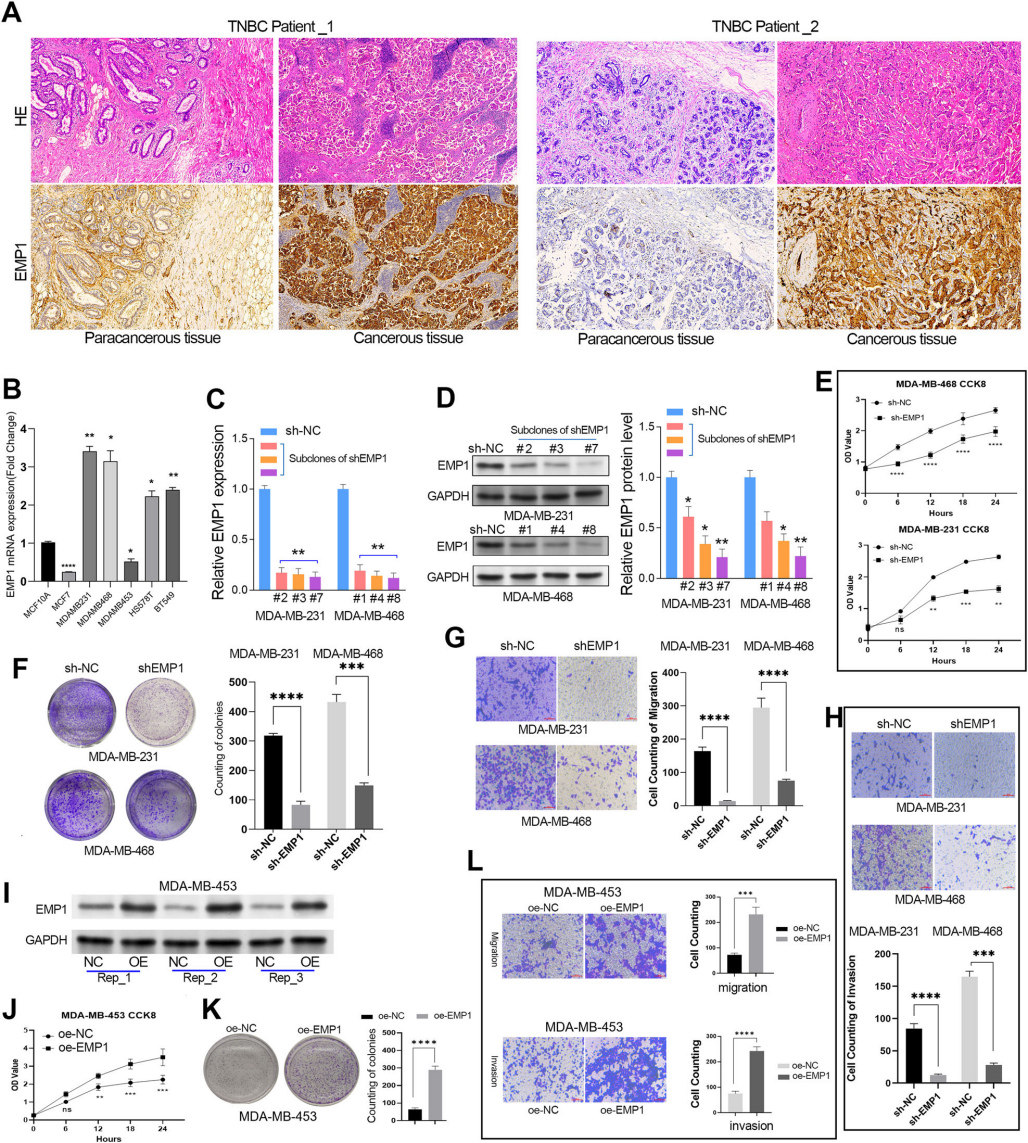
EMP1 exerts oncogenic properties in TNBC cell lines. **A** The HE staining and IHC assay (EMP1) from the same donor were verified in TNBC patients. The paracancerous tissue in the left panel (normal duct tissue) and in the right panel (normal lobules of breast tissue). IHC staining results showed that EMP1 expression was upregulated in TNBC tissues. **B** The expression pattern of EMP1 in different BC cell lines was determined using quantitative RT-PCR assay. **C**, **D** Multiple monoclonal cell lines with EMP1 knockdown were screened using quantitative RT-PCR and western blotting assays. **E**, **F** The CCK-8 and colony formation assays verified that EMP1 knockdown suppressed the proliferation of the TNBC cell lines. **G**, **H** The transwell migration and invasion assays showed that EMP1 knockdown significantly inhibited the migration and invasion of the TNBC cell lines. **I** EMP1 was overexpressed in TNBC cell line MDA-MB-453. **J**, **K** The CCK-8 and colony formation assays verified that EMP1 overexpression promoted the proliferation of the TNBC cells. **L** The transwell migration and invasion assays showed that EMP1 overexpression significantly facilitated the migration and invasion of the TNBC cells. **P < 0.01.

We determined EMP1 protein expression in IHC assays in paracancerous and cancerous TNBC tissues using IHC assays. The results showed that EMP1 was overexpressed in TNBC tissues, indicating that EMP1 may act as a pro-tumorigenic factor in TNBC (Fig. 3A). To test this hypothesis, gain- and loss-of-function studies of EMP1 were conducted in TNBC cell lines. According to the relative expression level of EMP1 mRNA in BC cell lines, EMP1 was highly expressed in the MDA-MB-231 and MDA-MB-468 cell lines, but was expressed at low levels in the MDA-MB-453 cell line (Fig. 3B). Thus, we selected the MDA-MB-453 cell line to establish an EMP1 overexpressing cell line and the MDA-MB-231 and MDA-MB-468 for the development of EMP1 knockdown cell lines. To achieve efficient and specific knockdown of EMP1, we constructed a 4-in-1 shRNA lentivirus targeting EMP1. After quantitative RT-PCR validation, we successfully obtained three subclones with EMP1 mRNA knockdown rates of 20–30% in two TNBC cell lines (Fig. 3C). Western blotting assays showed that the subclone #7 in the MDA-MB-231 cell line and the subclone #8 in the MDA-MB-468 cell line showed the most significant decrease in EMP1 protein levels (Fig. 3D). Therefore, we selected these two subclones to verify the effect of EMP1 depletion on the biological behavior of TNBC cells.

The subsequent CCK-8 assay indicated that knockdown of EMP1 obviously inhibited the growth rate of TNBC cell lines (Fig. 3E). Similarly, the colony formation assays confirmed that EMP1 knockdown significantly suppressed the cell proliferation in TNBC cell lines (Fig. 3F). Moreover, transwell migration and invasion assays together showed that EMP1 knockdown significantly decreased the abilities of migration and invasion of TNBC cell lines (Fig. 3G, H). Conversely, overexpression of EMP1 obviously promoted cell proliferation, migration, and invasion of the TNBC cell line MDA-MB-453 (Fig. 3I–L). These data together showed that EMP1 functioned as an oncogene in TNBC cells.

### EMP1 depletion inhibits TNBC proliferation and metastasis in vivo

**Fig. 4:**
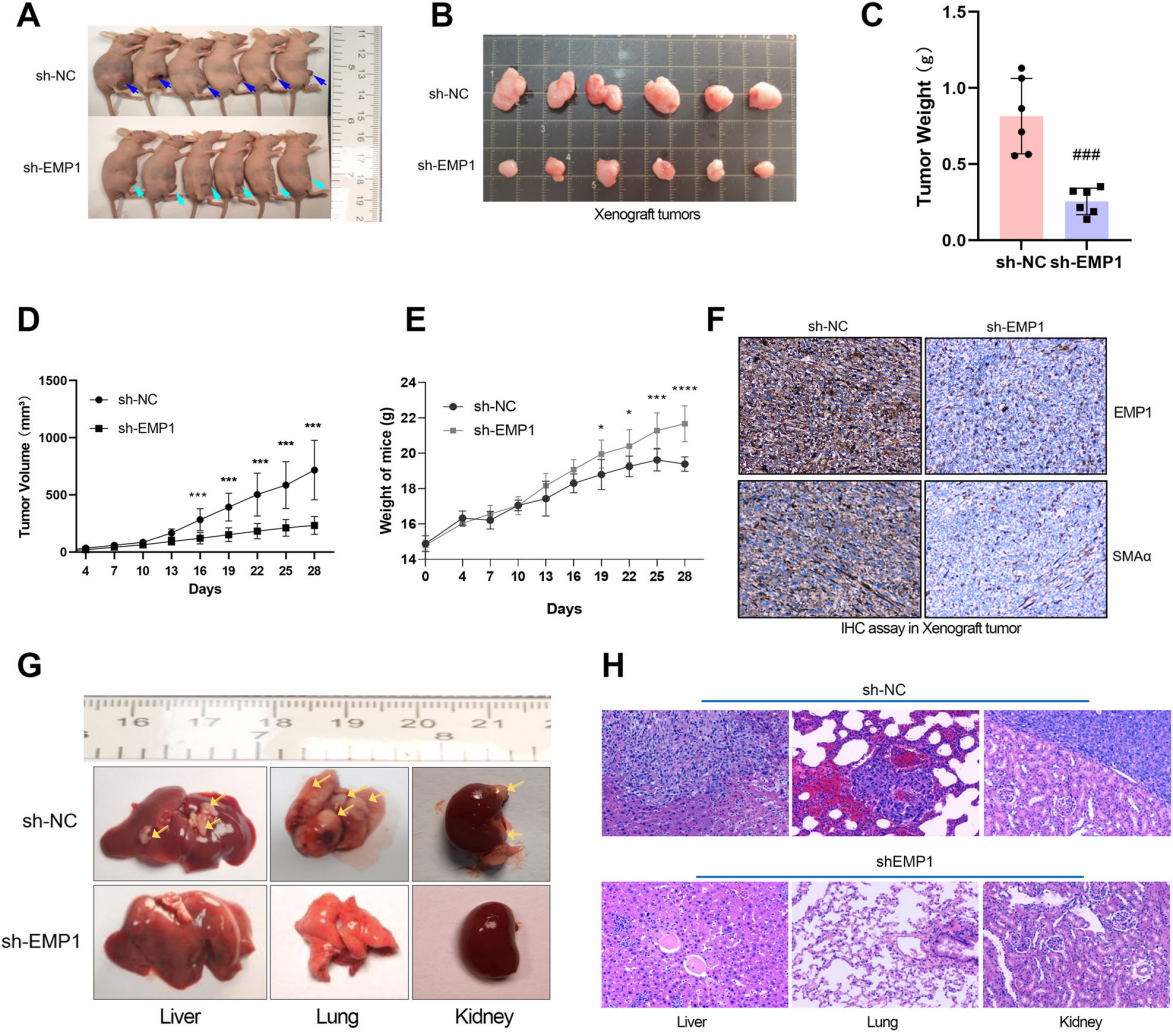
EMP1 depletion inhibited the proliferation and metastasis of xenograft tumors. **A** Morphology of tumor-bearing nude mice in the EMP1 knockdown and control groups. **B** Morphological differences in subcutaneous breast transplant tumors between the EMP1 knockdown and the control groups in nude mice. **C** The weight of transplanted tumors formed by TNBC cells in the EMP1 knockdown and control groups. **D** Volume growth curves of transplanted tumors between the EMP1 knockdown and the control groups. **E** Differences in body weight between the EMP1 knockdown group and the control group in nude mice. **F** Immunohistochemical staining results of EMP1 and αSMA in xenograft tumors in the EMP1 knockdown and control groups. **G** Observation of the number of metastatic foci formed by TNBC cells in the EMP1 knockdown and control groups in nude mice using a tail vein metastasis model. **H** Knockdown of EMP1 significantly inhibits the metastasis of TNBC cells in the liver, lung, and kidney organs of nude mice. **P < 0.01.

We investigated the biological role of EMP1 knockdown in vivo using a xenograft mouse model. Subcutaneous tumor-bearing and tail vein metastasis experiments in nude mice were performed in nude mice to investigate the effect of EMP1 knockdown on the growth of xenograft tumors and the hematogenous metastasis of TNBC cells. Equal numbers of TNBC cells with or without EMP1 knockdown were injected subcutaneously into the mammary gland of six female mice. After one month, we observed that the control group had larger xenograft tumors in the breast area than the EMP1 knockdown group (Fig. 4A). Subsequently, we dissected and weighed the xenograft tumors, and the results also showed that compared to the control group, the EMP1 knockdown group had a smaller size and lighter weight of the xenograft tumors (Fig. 4B, C). In addition, we found that EMP1 knockdown significantly reduced xenograft tumors volume (Fig. 4D). Consistently, the weight of mice bearing tumors formed by EMP1 knockdown TNBC cells was lower than that of mice in the control group (Fig. 4E). Compared to the control group, the transplanted tumors in the EMP1 knockdown group had lower CAF infiltration (Fig. 4F). This phenomenon was highly consistent with the clinical results and indicated that EMP1 plays an important role in CAF infiltration.

Because of the close correlation between metastasis and poor survival in TNBC patients, we used the tail vein metastasis model in nude mice to observe the effect of EMP1 knockdown on TNBC cell metastasizes. On the fortieth day after injection, the nude mice were euthanized, and the morphology of the lungs, kidneys, and spleen were evaluated. Significant metastases were observed in the lungs, kidneys, and spleen organs of the nude mice in the control group, whereas no visible metastases were observed in the EMP1 knockdown group (Fig. 4G). In addition, as shown in Fig. 4H, the size of distant metastases formed by EMP1 knockdown TNBC cells in the liver, lungs, and kidneys was smaller than that formed by the control group cells. These data indicated that EMP1 knockdown significantly inhibited the metastatic abilities of TNBC cells.

### EMP1 was required for the proliferation of CAFs in TNBC

**Fig. 5:**
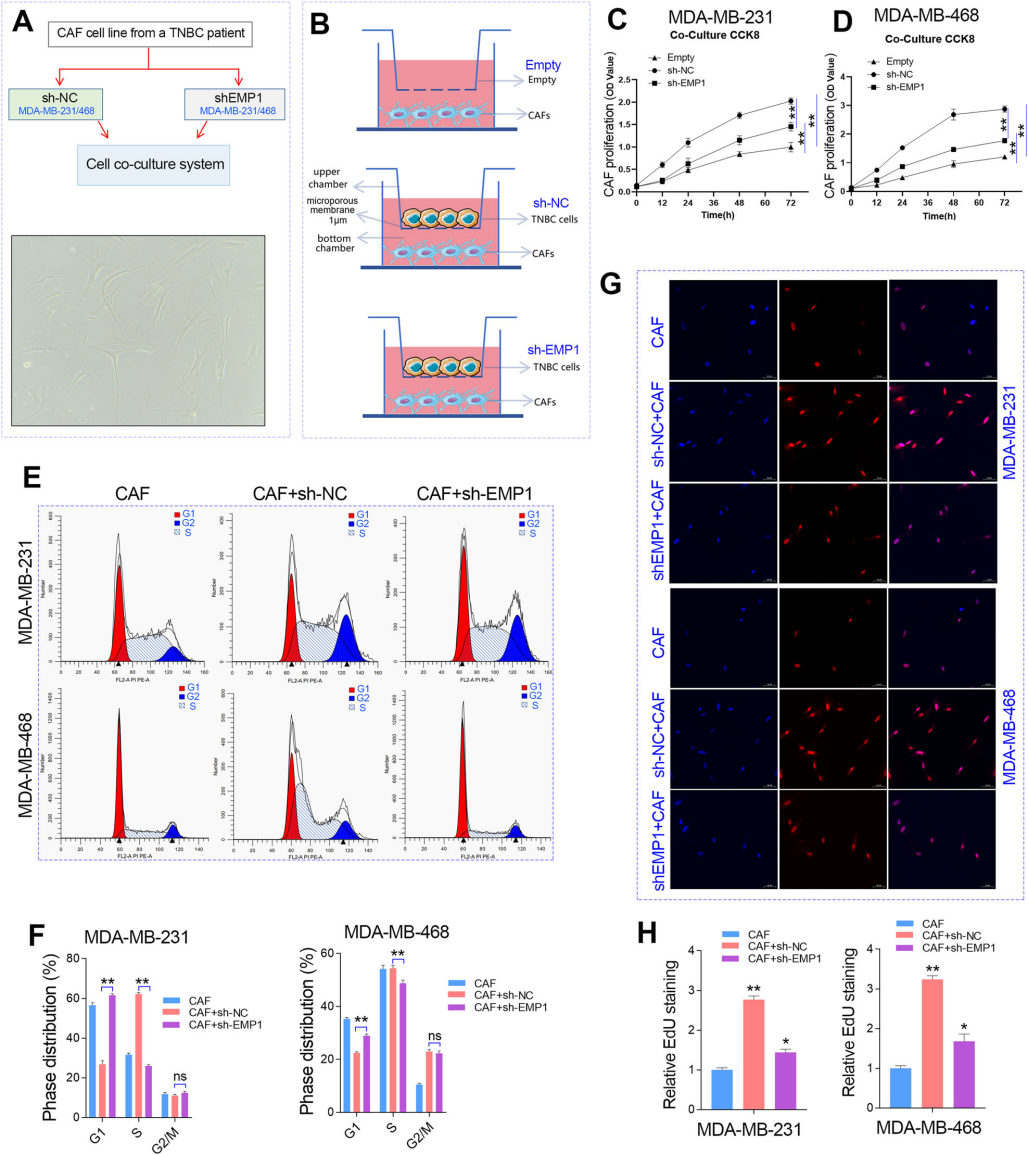
EMP1 knockdown in TNBC cells impaired the proliferation of CAFs. **A** Morphology of CAF cells isolated from the surgical resection tissue of a TNBC patient. **B** Schematic diagram of the cell co-cultivation system for different experimental groups, including CAF (empty), CAF+ sh-NC_TNBC cell line (sh-NC), and CAF+sh-EMP1_TNBC cell line (sh-EMP1). **C**, **D** The growth rate of CAFs in the cell co-cultivation system of different experimental groups was determined using the CCK-8 assay. **E**, **F** The cell cycle distribution of CAFs in the cell co-cultivation system of different experimental groups was determined by flow cytometry. **G**, **H** The cell proliferation rate of CAFs in the cell co-cultivation system of different experimental groups was determined using the EdU assay. *P < 0.05; **P < 0.01.

To understand the role of EMP1 in CAF infiltration in TNBC, we first established a CAF cell line from a TNBC patient who has underwent tumor resection surgery and cell co-cultured the cells with TNBC cell lines. The morphology of the CAFs under a light microscope is shown in Fig. 5B. The experimental design of the co-culture system is shown in Fig. 5C, and can be divided into three groups: CAF alone, CAF co-culture with wild-type TNBC cells (sh-NC), and CAF co-culture with EMP1 knockdown TNBC cells (sh-EMP1). We then determined the proliferative ability of CAFs using CCK-8, EdU, and cell cycle distribution assays. CCK-8 assays showed that the growth rate of the CAFs co-cultured with sh-NC TNBC cell lines was higher than that of CAF alone, but lower than that of CAFs co-cultured with sh-EMP1 TNBC cells (Fig. 5D). The cell cycle assay further confirmed that EMP1 knockdown in TNBC cell lines disrupted the G1/S transition of CAFs (Fig. 5E, F). Similarly, the EdU assays showed that EMP1 knockdown in TNBC cell lines suppressed the proliferation rate of CAFs (Fig. 5G, H). These results provide direct evidence that EMP1 plays a role in the cell-cell communication between TNBC cells and CAFs.

### EMP1 knockdown in TNBC cells impedes CAF infiltration in xenografts

**Fig. 6:**
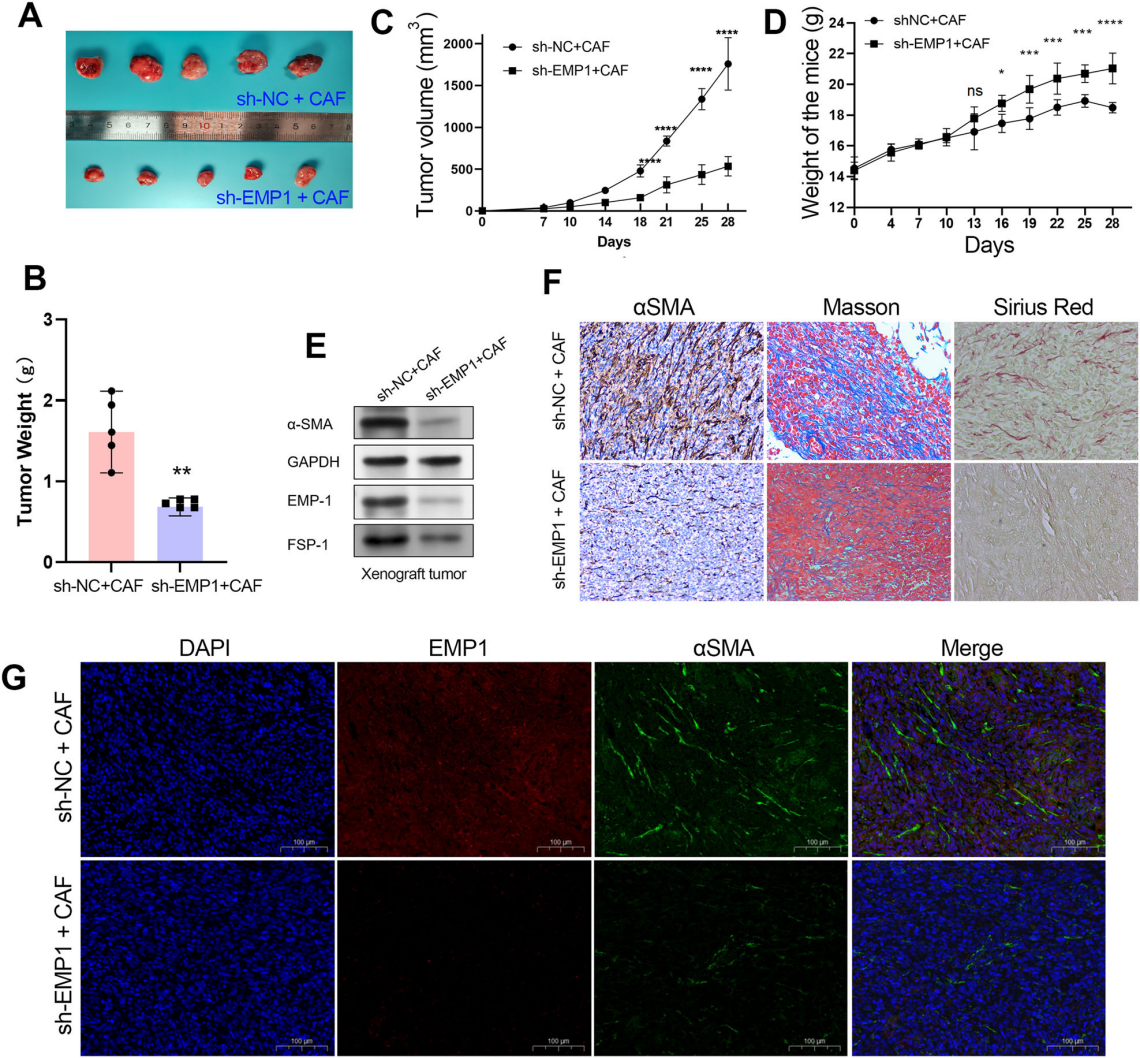
EMP1 was required for CAF infiltration in xenografts. **A**, **B** A mixture of TNBC cells and CAFs was injected subcutaneously into the mammary gland of nude mice, and the morphology and weight of the xenograft tumors formed by TNBC cells are shown in the plots. **C** Tumor volume growth curve of xenograft tumors in the CAF+ sh-NC_TNBC cell line (sh-NC) and the CAF+ sh-EMP1_TNBC cell line (sh-EMP1) groups. **D** The body weight of mice in the CAF+ sh-NC_TNBC cell line (sh-NC) group and the CAF+ sh-EMP1_TNBC cell line (sh-EMP1) group. **E** The expression of CAF biomarkers in xenograft tumors was determined by western blotting assay. **F** The CAF infiltration level in xenograft tumors is examined by IHC, Masson, and Sirius Red staining assays. **G** Knockdown of EMP1 in TNBC cells significantly inhibited CAF infiltration in xenograft tumors. Multicolor immunofluorescence experiments were performed in the xenograft tumors between sh-NC + CAF group and sh-EMP1 + CAF group. The CAFs are labeled by fibroblast biomarker anti-α-SMA with green fluorescence. The EMP1 expression in xenograft tumors was evaluated using anti-EMP1 bodies with red fluorescence. **P < 0.01.

To further evaluate the biological effects of EMP1 on CAF infiltration in vivo, we resuspended TNBC cells and CAFs at a 5:1 ratio and injected the cells into a nude mouse xenograft tumor model. Similarly to our previous observation, the xenograft tumors in the EMP1 knockdown group were smaller and lighter than those in the control group (Fig. 6A–C). Furthermore, mice in the EMP1 knockdown group had heavier than the xenograft tumors than those in the control group (Fig. 6D). We conducted western blotting, IHC, and multicolor immunofluorescence experiments on the xenograft tumors to confirm the biological effects of EMP1 on CAF infiltration. The results showed that the xenograft tumors in the EMP1 knockdown group had an obvious decrease in CAF infiltration level compared to those in the control group (Fig. 6E–G). These data together confirmed that EMP1 plays an essential role in regulating the crosstalk between TNBC cells and CAFs.

### EMP1 knockdown impaired NF-κB signaling pathways in TNBC cell lines

**Fig. 7:**
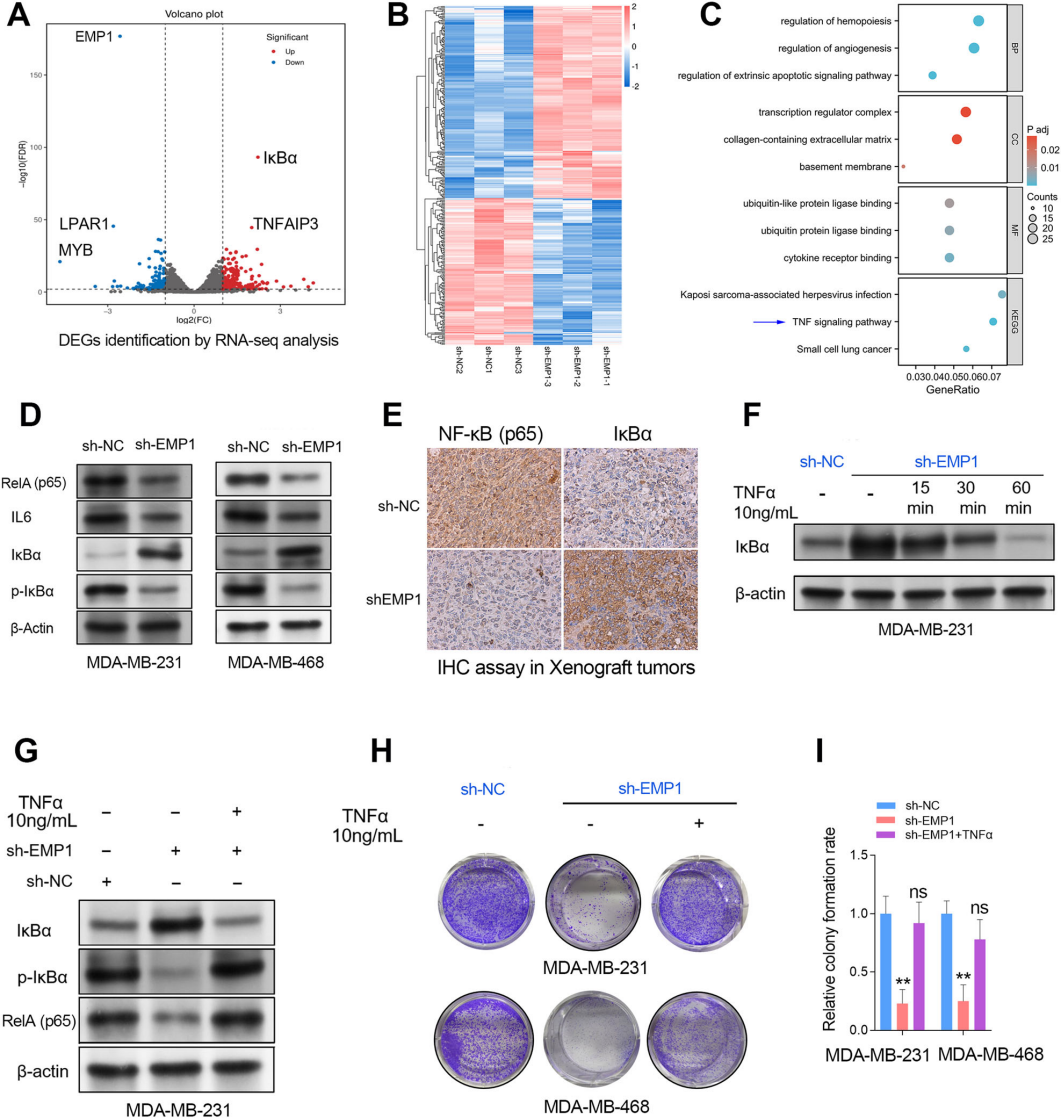
EMP1 knockdown inhibited TNBC progression through the NF-κB signaling pathway. **A**, **B** The RNA-seq studies were conducted in the MDA-MB-231 cell lines with or without EMP1 knockdown. The DEGs after EMP1 depletion were shown in the volcano map and heat plot. **C** The GO/KEGG analysis was performed using the DEGs from the EMP1 knockdown experiment. **D** Western blotting assay confirmed that EMP1 knockdown activates the NF-κB/IL6 axis in TNBC cell lines. **E** The IHC assay of RelA/p65 and IκBα was conducted in xenograft tumors formed by TNBC cell lines with or without EMP1 knockdown. **F** The expression level of IκBα can be restored by recombinant active TNFα protein (10 ng/mL). **G** The rescue western blotting assay confirmed that EMP1 knockdown inhibited NF-κB signaling through upregulating expression of IκBα. **H, I** The rescue colony formation assay verified that EMP1 knockdown inhibited TNBC cell proliferation through IκBα/NF-κB signaling.

To understand the molecular mechanisms by which EMP1 mediates the crosstalk between TNBC cells and CAFs, we conducted RNA sequencing (RNA-seq) studies in the TNBC cell line MDA-MB-231. The differentially expressed genes (DEGs) after EMP1 knockdown are shown in Fig. 7A, B and include IκBα (NFKBIA), a well-known inhibitor of NF-κB signaling. We then collected these DEGs for GO/KEGG analysis. The results showed that the DEGs produced by EMP1 depletion were enriched in the TNF-α-mediated inflammation signaling pathway (Fig. 7C). Therefore, we evaluated the effects of EMP1 depletion on the NF-κB signaling in TNBC cell lines using western blotting assays. The results showed that EMP1 depletion obviously increased the expression of IκBα and decreased the expression of NFκB (RelA or p65), phosphorylated IκBα, and IL6 (a downregulated target of NF-κB signaling) in TNBC cell lines (Fig. 7D). In addition, we also observed a similar phenomenon in xenograft tumors: NF-κB1 was downregulated, while IκBα protein was upregulated in the xenograft tumor formed by the EMP1 knockdown TNBC cells (Fig. 7E). Then, we further conducted a rescue assay using exogenous human recombinant TNFα protein (10 ng/mL) to verify whether EMP1 knockdown inhibits TNBC progression via NF-κB signaling. The results demonstrated that TNFα protein treatment could obviously reduce the expression level of IκBα protein in the wild-type and sh-EMP1 MDA-MB-231 cells (Fig. 7F, G). In addition, the rescue cell colony formation assay further confirmed that EMP1 knockdown inhibited TNBC cell proliferation through the NF-κB signaling pathway (Fig. 7H, I).

### EMP1 knockdown inhibited CAF infiltration through suppressing IL6 secretion in TNBC cells

**Fig. 8:**
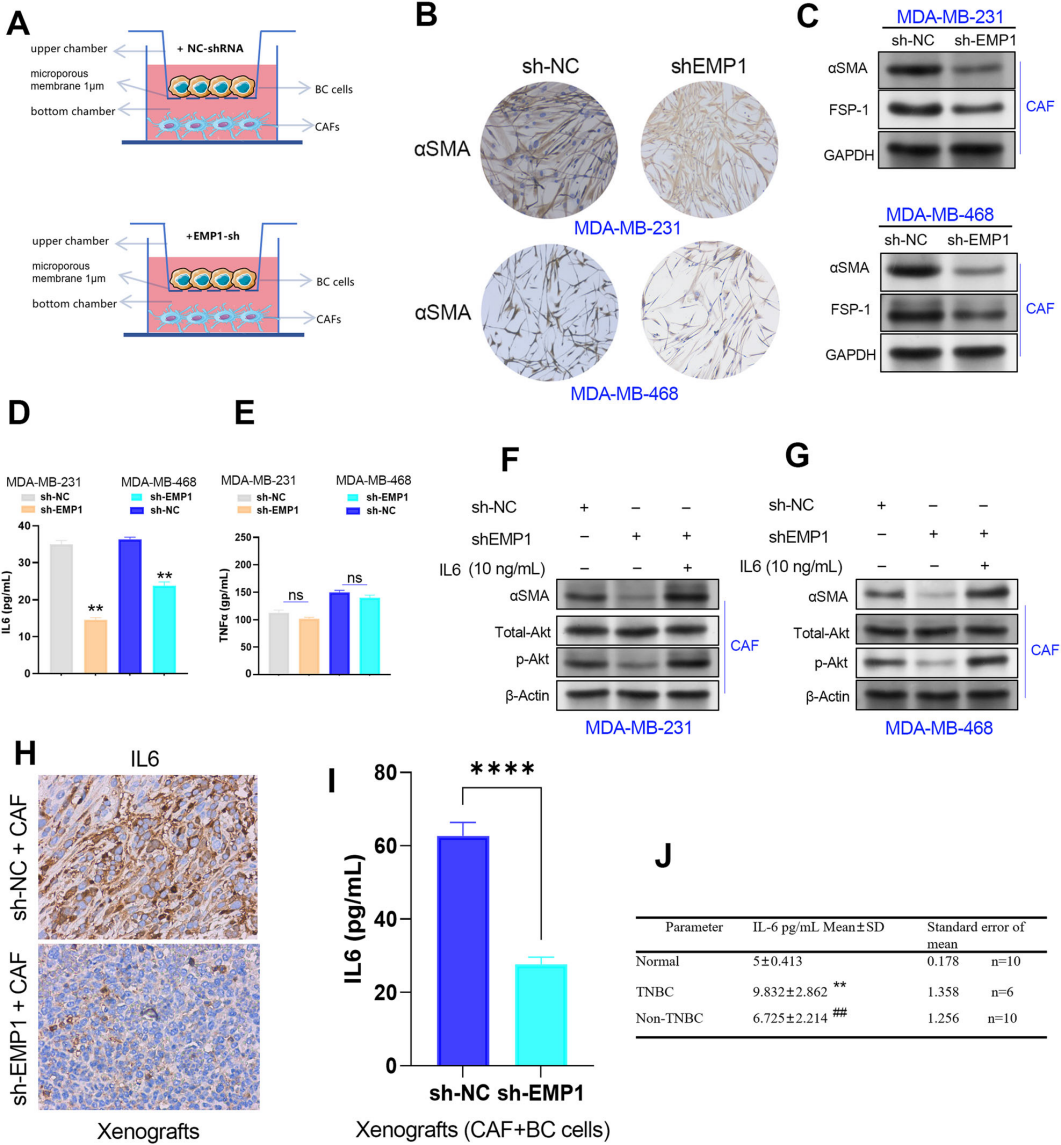
EMP1 promoted CAF infiltration by enhancing IL6 secretion in TNBC cells. **A** The microporous membrane of the cell co-culture system only allows small molecules, such as secreted proteins, to pass through, and cells are too large to pass through the membrane. **B** EMP1 knockdown in TNBC cells suppressed the expression of αSMA in CAFs co-cultured with the corresponding TNBC cell lines. **C** EMP1 knockdown in TNBC cells suppressed the expression of αSMA and FSP-1 in CAFs co-cultured with corresponding TNBC cell lines. **D** ELISA assay confirmed that EMP1 knockdown decreased IL6 secretion in TNBC cell lines. **E** ELISA assay confirmed that EMP1 knockdown had no obvious effect on TNFα secretion in TNBC cell lines. **F**, **G** Recombinant active IL6 protein (10 ng/mL) rescued αSMA expression and increased active Akt expression in CAFs. **H** The IL6 protein level in xenograft tumors formed by TNBC cell lines with or without EMP1 knockdown was determined using an IHC assay. **I** The IL6 level in xenograft blood between mice in the EMP1 knockdown and the control groups were determined using ELISA. **J** The IL6 level in the blood of healthy volunteers (*N* = 10), non-TNBC breast cancer patients (*N* = 10), and TNBC patients (*N* = 6).

Our next goal was to elucidate the molecular mechanism of EMP1 in the crosstalk between TNBC cells and CAFs. From the results of the cell co-culture system, it was evident that knocking down EMP1 in TNBC cells significantly reduces the expression level of CAF biomarkers (αSMA, FSP-1) in CAFs (Fig. 7A–C). Owing to the small pore size of the microporous membrane (1 μm) in the co-culture system only the secreted proteins were allowed to pass through. Therefore, we evaluated the inflammation-relevant secretory factors, including IL6 and TNFα, in TNBC cell lines using ELISA. The results showed that EMP1 depletion significantly reduced the expression level of IL6 in TNBC cell lines, but did not decrease the expression level of TNFα (Fig. 8D, E).

We speculated that EMP1 knockdown may impair the secretion of IL6, resulting in the inhibition of CAF proliferation. To verify this possibility, we conducted a rescue assay using exogenous human recombinant IL6 active protein in a co-culture system. The rescue assay confirmed that recombinant IL6 active protein could recover the reduced level of αSMA protein in CAFs caused by EMP1 knockdown in TNBC cell lines (Fig. 8F, G). Consistent with our speculation, the IHC staining results showed that IL6 protein level in the sh-EMP1+CAF group were lower than those in the control group (Fig. 8H). Moreover, EMP1 knockdown decreased IL6 protein level in mouse blood (Fig. 8I). In addition, we also determined the expression levels of IL6 in the blood of different subtypes of BC patients as well as healthy volunteers using the ELISA method. Consistently, the IL6 protein level in the blood of TNBC patients were higher than those in the blood of non-TNBC patients (Fig. 8J).

**Fig. 9:**
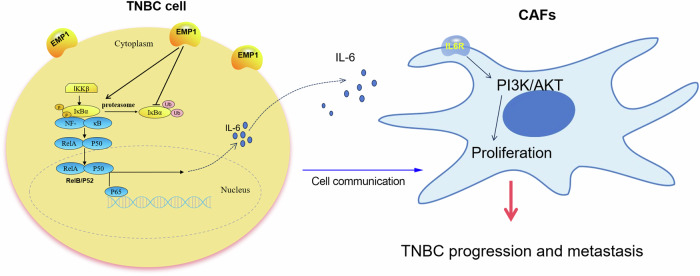
EMP1 promotes TNBC progression and metastasis by mediating cell-cell communication between cancer cells and CAFs and enhancing CAF infiltration.

In conclusion, based on our results, we proposed a working model for the carcinogenic effects of EMP1 in TNBC (Fig. 9). Briefly, EMP1 is highly expressed in TNBC cells, where it enhances IL6 secretion through the NF-κB signaling pathway. Meanwhile, TNBC cell-derived IL6 promotes CAF proliferation by increasing the levels of activated AKT, thereby promoting TNBC progression and metastasis.

## Discussion

The secretome in the TME is composed of various secretory proteins from all the cells in the TME, including CAF, immune cells, and tumor cells. Autocrine and paracrine actions of these secreted proteins play a very important role in cell communication. Cell communication between cancer cells and CAFs should be a bidirectional multipath network. However, research on the interaction between CAF and tumor cells has mainly focused on the effects of CAF on tumor cells, while the biological effects of tumor cells on CAF cells remain poorly understood.

In this study, we provided evidence that tumor cell-derived secreted proteins can also participate in the proliferation of CAFs. Similar to our view, Thompson and colleagues have reported that pancreatic cancer-derived cytokines promote the inflammatory fibroblast phenotype under hypoxic conditions [48]. In addition, Giusti et al. have reported that tumor-derived extracellular vesicles promote CAF infiltration and sustain a pro-tumorigenic microenvironment [49].

In the present study, we have comprehensively evaluated the infiltration landscape of immune cells and stromal cells in different subtypes of BC by deconvolution analysis of the bulk RNA-seq data in the TCGA_BRCA cohort using the xCell algorithm. We discovered a critical role for EMP1 in TME remodeling of BC, especially in triple-negative breast cancer (TNBC). The joint results of bioinformatics analysis and IHC staining based on continuous pathological slices confirmed that EMP1 expression was closely correlated with CAF infiltration in BC (Figs. 1 and 2). Notably, EMP1 and αSMA (a classic biomarker in CAF) were highly co-expressed and predicted a poor prognosis in different subtypes of BC.

According to the seed-and-soil theory, the occurrence and development of tumors results from the mutual influence and co-evolution between tumor cells (seeds) and TME (soil) [16, 18, 24]. CAFs are important components of the TME and have been shown to be closely associated with cancer proliferation, progression, and metastasis [50]. In BC, CAF are considered the key regulator of the breast cancer TME and a multifaceted driver of BC progression [51, 52]. Previous studies have shown that CAFs usually exert their functions in remodeling the extracellular matrix, cellular metabolism, and cell-cell communications by secreting cytokines, exosomes, and other signaling molecules [17]. For example, CAF-secreted TGF-β promotes BC metastasis by activating epithelial-mesenchymal transition (EMT) signaling [53, 54]. Furthermore, CAF-derived LRRC15 was found to promote TNBC cell migration and invasion via the Wnt/β‑catenin signaling pathway [55]. In addition, CAF-derived exosomes suppress BC progression through immune evasion mediated by PD-L1 [56].

Our current data strongly implies that EMP1 might play a role in CAF infiltration in BC. Considering that EMP1 is an epithelial membrane protein, it is primarily expressed in epithelial cells. The correlation between EMP1 and CAF infiltration may be because of the cell communication between epithelial cells and CAFs. Therefore, we have herein introduced a cell co-culture system to validate the role of EMP1 in mediating the cell communication between epithelial cells and CAFs. On one hand, through gain-of-function and loss-of-function studies, we confirmed that EMP1 positively regulates TNBC cell proliferation, migration, and invasion in vivo and in vitro via activation of NF-κB signaling. On the other hand, we found that EMP1 is essential for mediating communication between TNBC cells and CAFs through in vivo and in vitro experiments. More importantly, our cell co-culture experiments strongly indicated that the cell communication between TNBC cells and CAFs mediated by EMP1 was dependent on TNBC cell-derived secretory proteins.

As we have confirmed that EMP1 knockdown had a profound effect on the NF-κB signaling pathway in TNBC cells, we measured the expression levels of inflammation-related secretory factors in vivo and in vitro. Through rescue experiments, we ultimately confirmed that EMP1 mediated the interaction between TNBC cells and CAFs by regulating the secretion of IL6 in TNBC cells, thereby promoting CAF proliferation and infiltration, and thereby mediating the progression and metastasis of TNBC (Fig. 9). Our study highlights that EMP1 can serve as a therapeutic target for TNBC patients by inhibiting metastasis through antagonizing CAF infiltration.

## Supplementary information

Supplementary Table S1

Supplementary Table S2

Original WB images

## Data Availability

Data are available upon reasonable request. All data relevant to the study are included in the article or uploaded as online supplemental information.
