# Supplementary material for: Tumor cell-derived EMP1 is essential for cancer-associated fibroblast infiltration in tumor microenvironment of triple-negative breast cancer
Source: Cell Death Dis. 2025 Feb 27;16(1):143. doi: 10.1038/s41419-025-07464-9 (PMC11868485; doi:10.1038/s41419-025-07464-9)
Supplement: Supplementary file 1 — Supplementary Table S1 [file 41419_2025_7464_MOESM1_ESM.docx]

**Table S2.** The clinical pathology information of BC patients enrolled in this study.

| Characteristics | Lumina A  (N=14) | Lumina B (N=16) | HER2+ (N=15) | TNBC (N=29) |  |
| --- | --- | --- | --- | --- | --- |
| N | 14 (18.9%) | 16 (21.6%) | 15 (20.3%) | 29 (39.2%) |  |
| Age Group | | | | | |
| <60 | 6 (42.9%) | 9 (56.3%) | 5 (33.3%) | 19 (65.5%) |  |
| ≥60 | 8 (57.1%) | 7 (43.8%) | 10 (66.7%) | 10 (34.5%) |  |
| Tumor Characteristics | | | | | |
| Tumor Location | | | | | |
| Left | 8 (57.1%) | 7 (43.8%) | 6 (40.0%) | 15 (51.7%) |  |
| Right | 6 (42.9%) | 9 (56.3%) | 9 (60.0%) | 14 (48.3%) |  |
| Histological Type | | | | | |
| Ductal carcinoma | 10 (71.4%) | 9 (56.3%) | 10 (66.7%) | 20 (69.0%) |  |
| Lobular carcinoma | 2 (14.3%) | 3 (18.8%) | 2 (13.3%) | 4 (13.8%) |  |
| Grade | | | | | |
| Grade I | 3 (21.4%) | 2 (12.5%) | 1 (6.7%) | 0 |  |
| Grade II | 7 (50.0%) | 8 (50.0%) | 6 (40.0%) | 8 (27.6%) |  |
| Grade III | 4 (28.6%) | 6 (37.5%) | 8 (53.3%) | 19 (72.4%) |  |
| Tumor Size (pT Stage) | | | | | |
| T2 | 10 (71.4%) | 8 (50.0%) | 5 (33.3%) | 12 (41.4%) |  |
| T3 | 3 (21.4%) | 6 (37.5%) | 8 (53.3%) | 15 (51.7%) |  |
| T4 | 1 (7.1%) | 2 (12.5%) | 2 (13.3%) | 2 (6.9%) |  |
| Number of Nodes (pN Stage) | | | | | |
| N0 | 4 (28.6%) | 3 (18.8%) | 2 (13.3%) | 2 (6.9%) |  |
| N1 | 6 (42.9%) | 7 (43.8%) | 5 (33.3%) | 12 (41.4%) |  |
| N2 | 2 (14.3%) | 4 (25.0%) | 5 (33.3%) | 7 (24.1%) |  |
| N3 | 2 (14.3%) | 2 (12.5%) | 3 (20.0%) | 8 (27.6%) |  |
| Number of Positive Nodes | | | | | |
| Positive Nodes | 3 (21.4%) | 5 (31.3%) | 4 (26.7%) | 10 (34.5%) |  |
| Metastatic Status (pM Stage) | | | | | |
| M0 | 13 (92.9%) | 12 (75.0%) | 10 (66.7%) | 15 (51.7%) |  |
| M1 | 1 (7.1%) | 4 (25.0%) | 5 (33.3%) | 14 (48.3%) |  |
| St Gallen consensus Biomarkers | | | | | |
| ER Status | Positive (14) | Positive (16) | Positive (0) | Positive (0) |  |
| PR Status | Positive (14) | Positive (5) | Positive (0) | Positive (0) |  |
| HER2 Status | Negative (0) | Positive (3) | Positive (15) | Negative (0) |  |
| Ki-67 Index | <14% (14) | ＜14% (0) | >14% (15) | >14% (29) |  |
