# Supplementary material for: Tumor cell-derived EMP1 is essential for cancer-associated fibroblast infiltration in tumor microenvironment of triple-negative breast cancer
Source: Cell Death Dis. 2025 Feb 27;16(1):143. doi: 10.1038/s41419-025-07464-9 (PMC11868485; doi:10.1038/s41419-025-07464-9)

Figure 3C

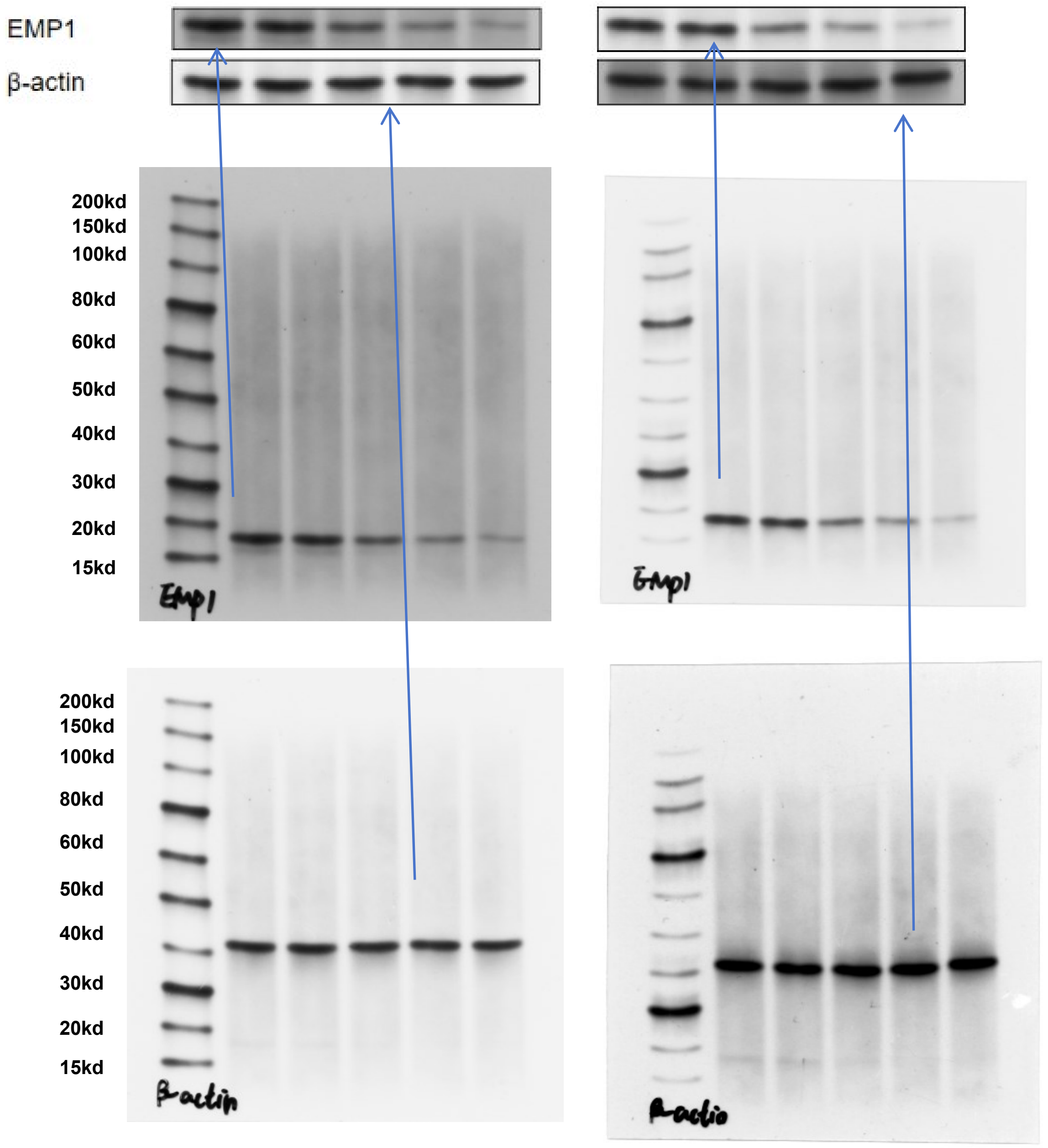

EMP1
β-actin
200kd
150kd
100kd
80kd
60kd
50kd
40kd
30kd
20kd
15kd
EMP1
EMP1
200kd
150kd
100kd
80kd
60kd
50kd
40kd
30kd
20kd
15kd
β-actin
β-actin

Figure 6E

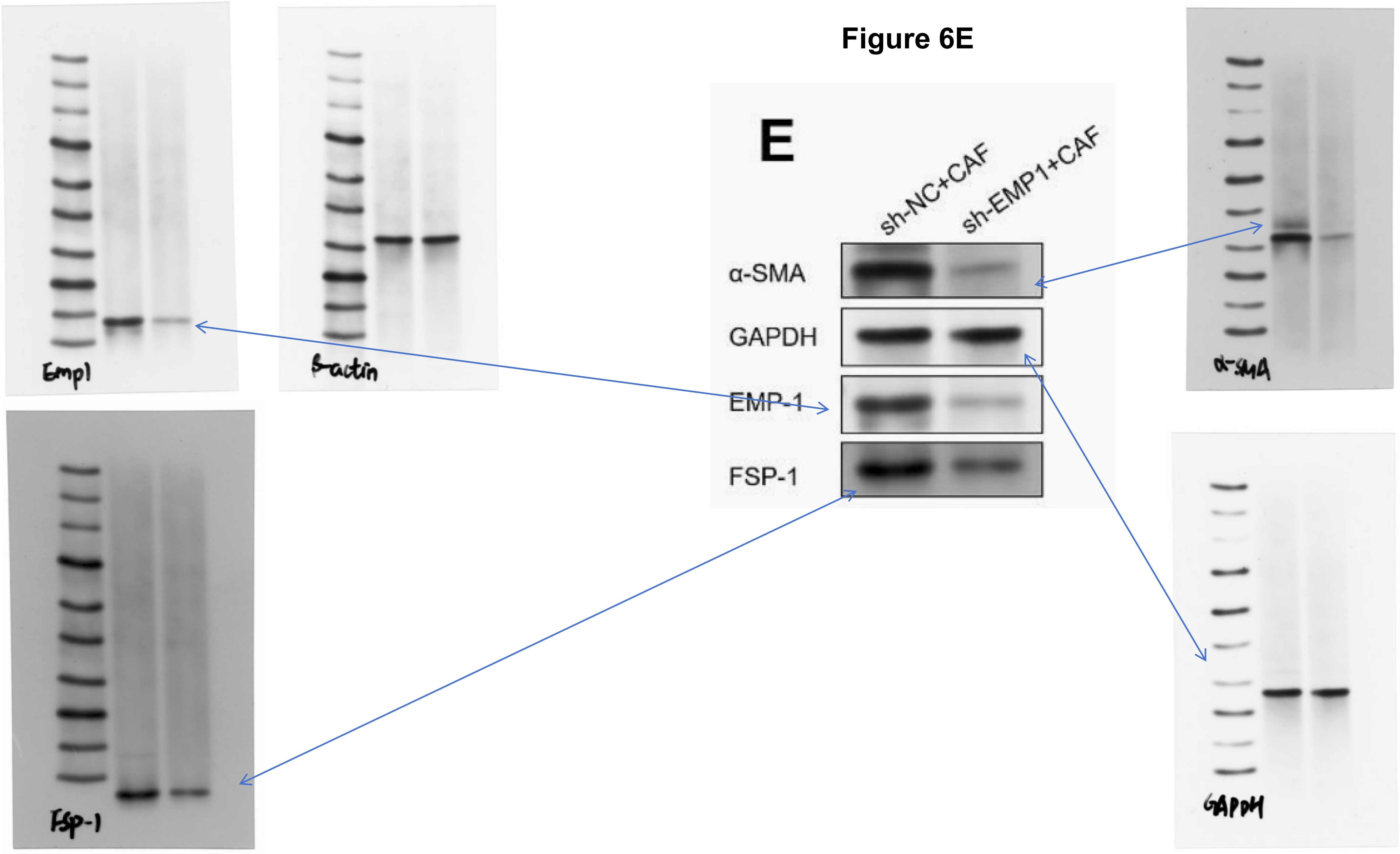
E
sh-NC+CAF
sh-EMP1+CAF
α-SMA
GAPDH
EMP-1
FSP-1
Empl
β-actin
Fsp-1
α-SMA
GAPDH

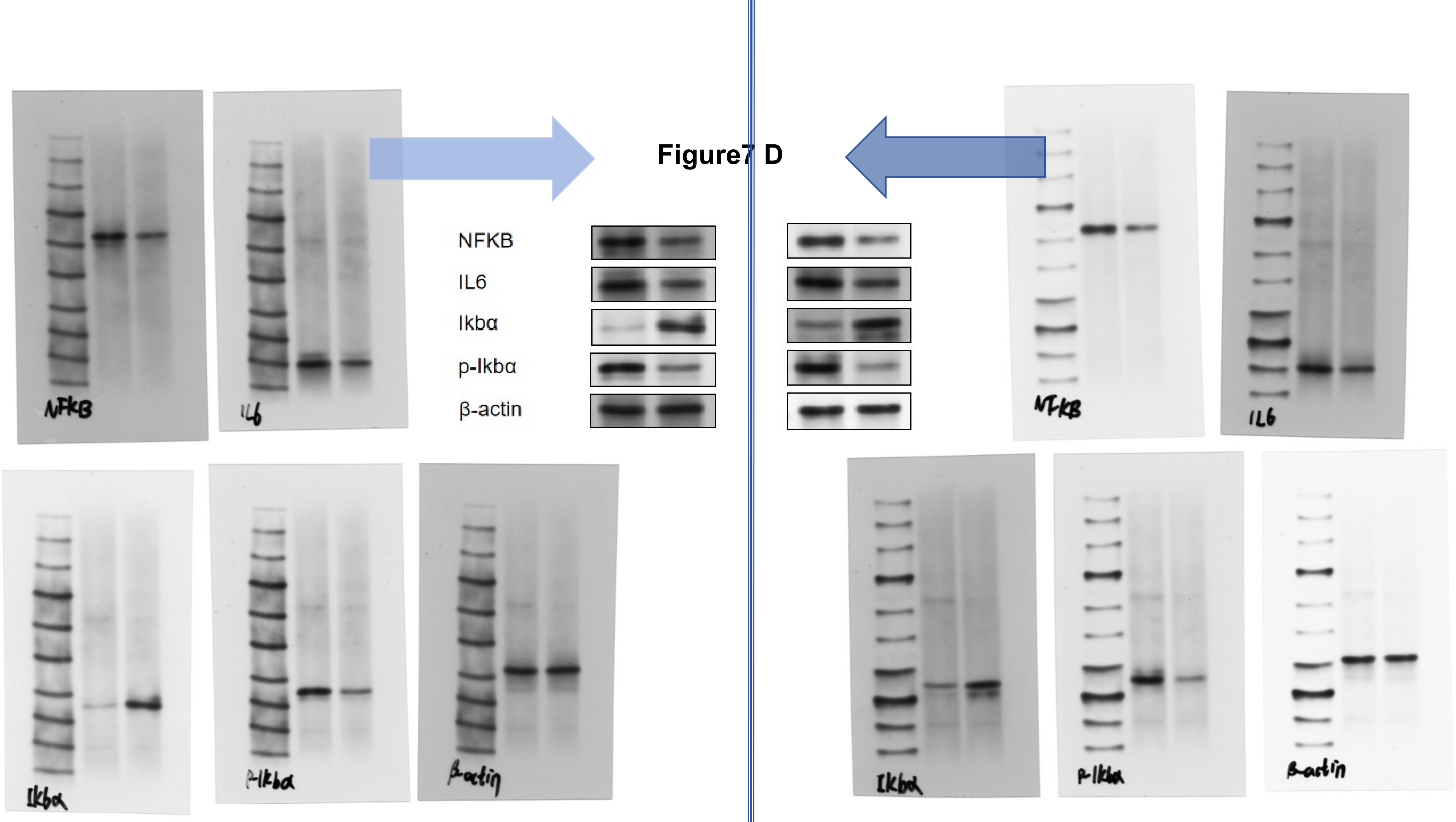
Figure7 D
NFKB
IL6
Ikbα
p-Ikbα
β-actin
NFKB
IL6
Ikbα
p-Ikbα
β-actin
NFKB
IL6
Ikbα
p-Ikbα
β-actin

Figure7 F

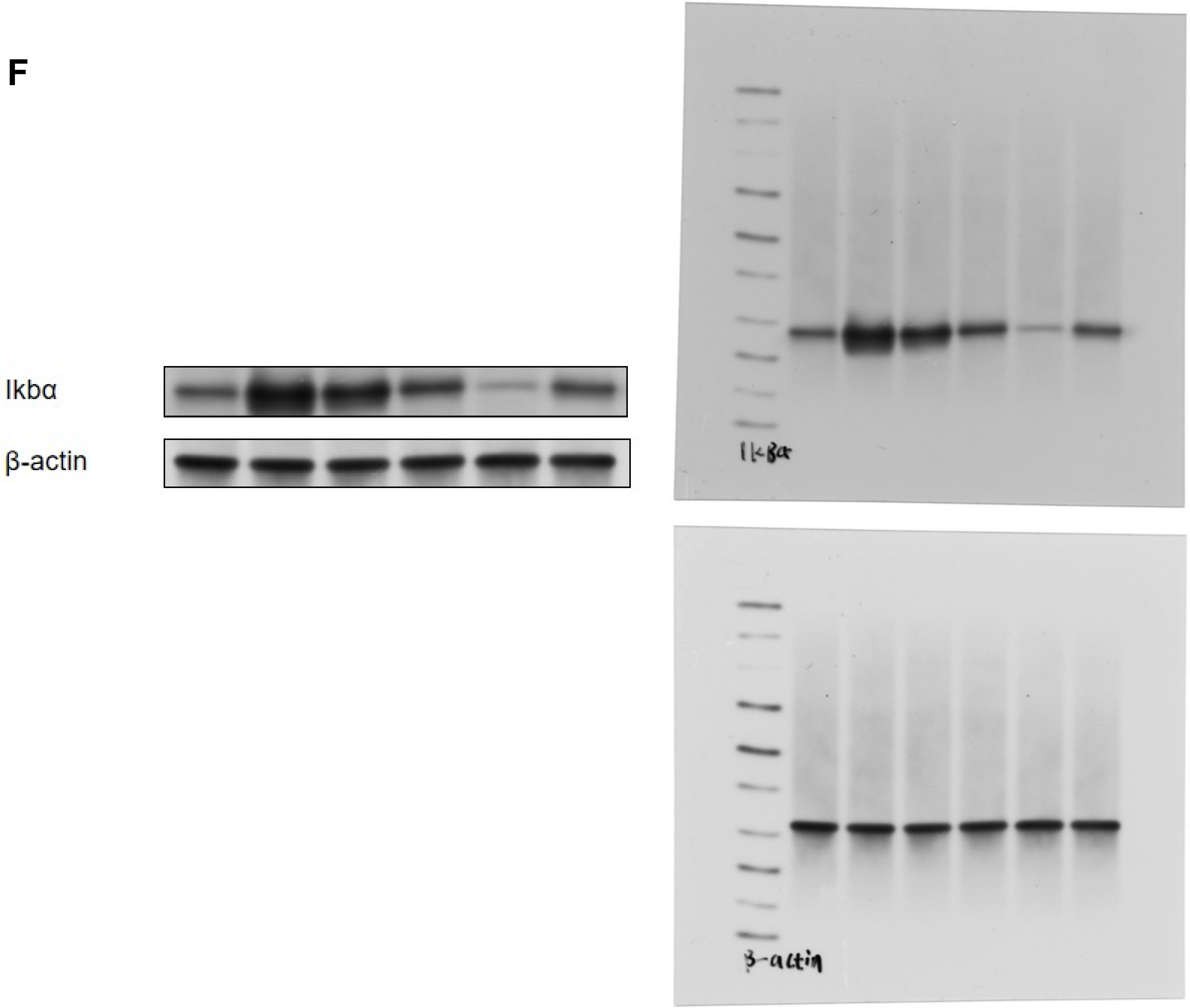

Figure7 G

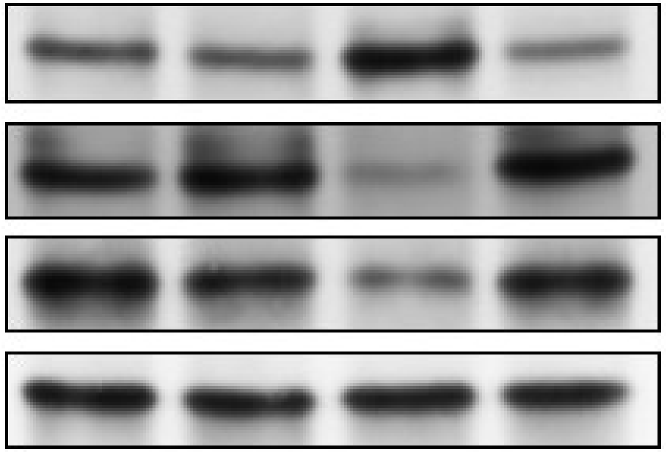

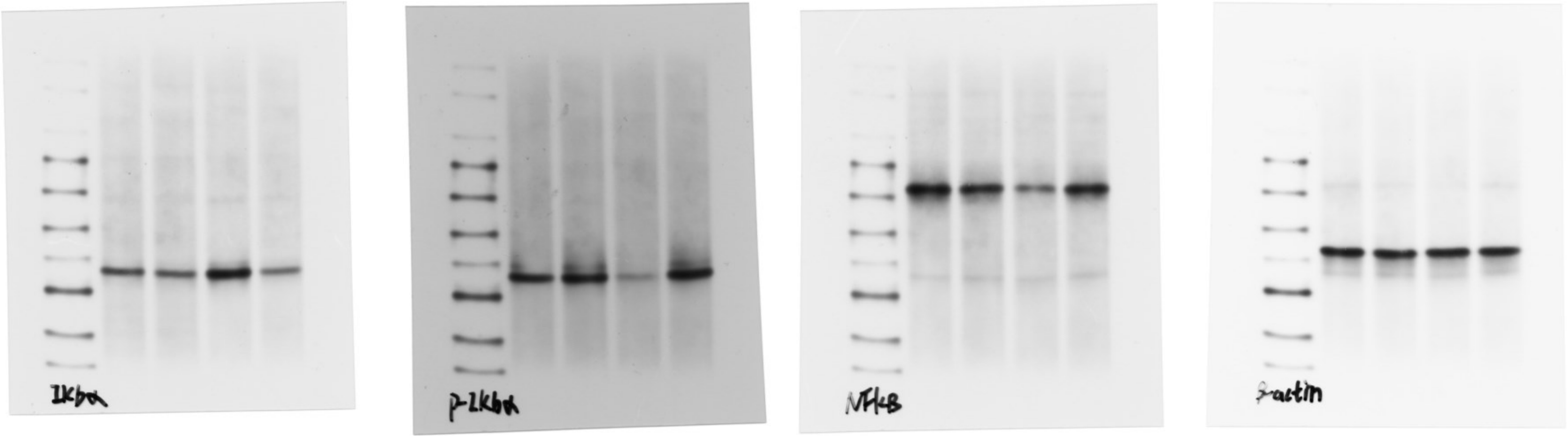

Figure8 C

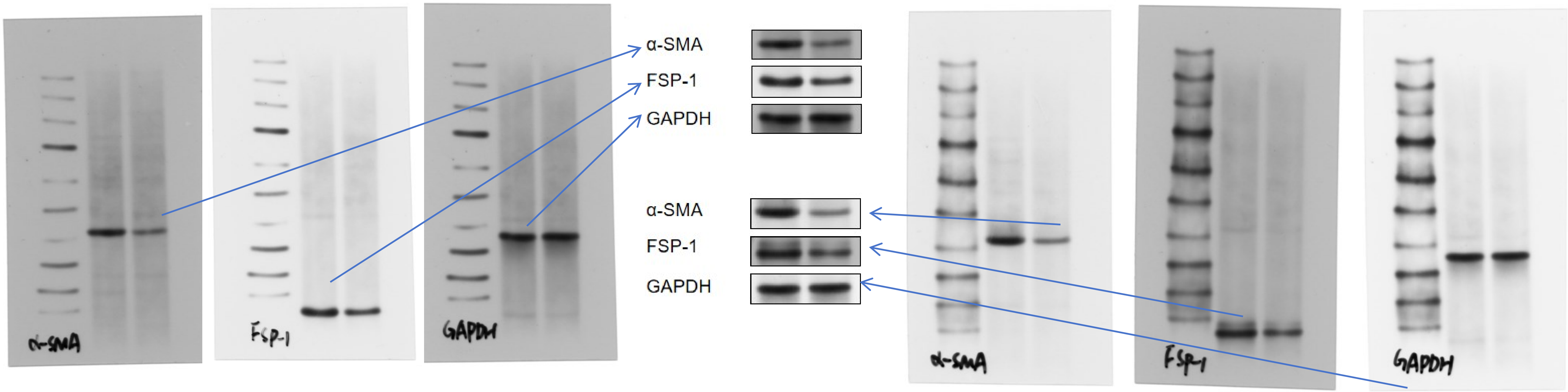

Figure8 F

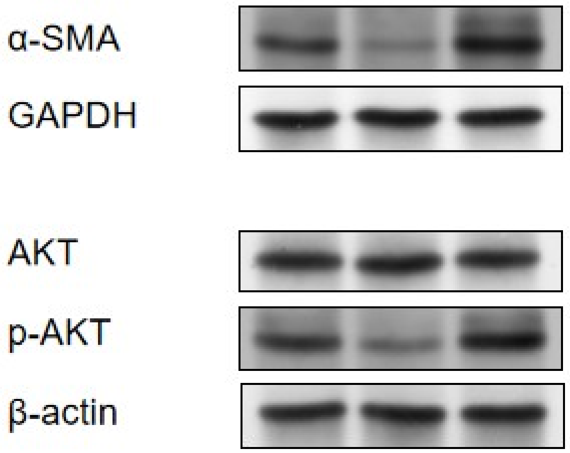

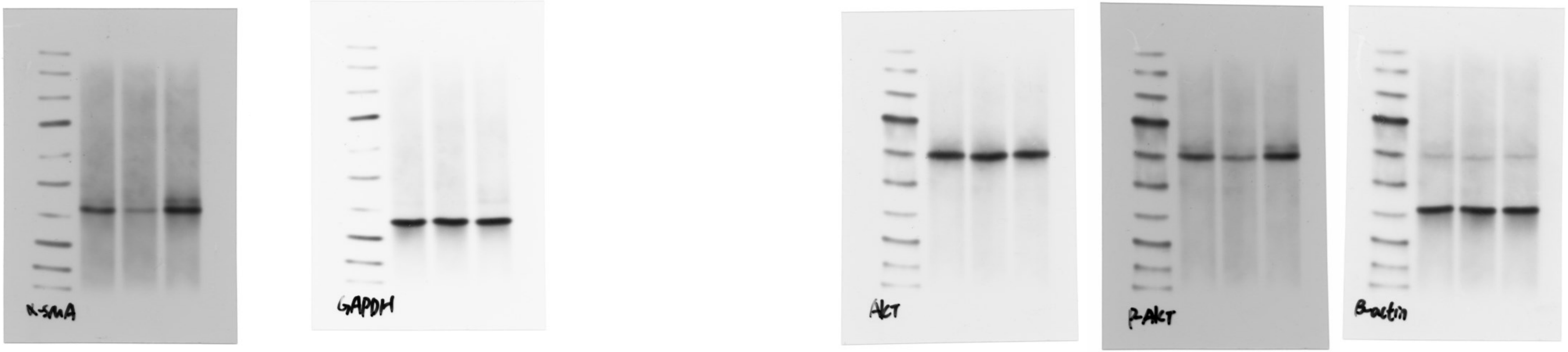

Figure8 G

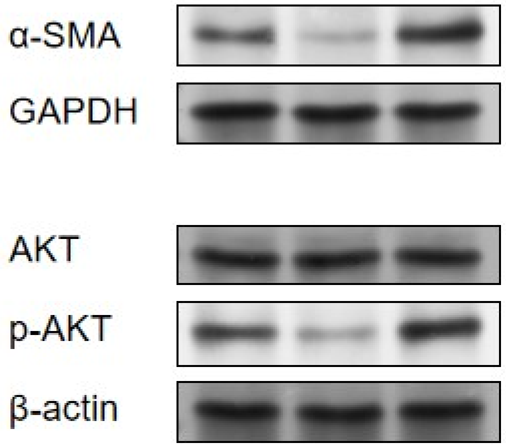

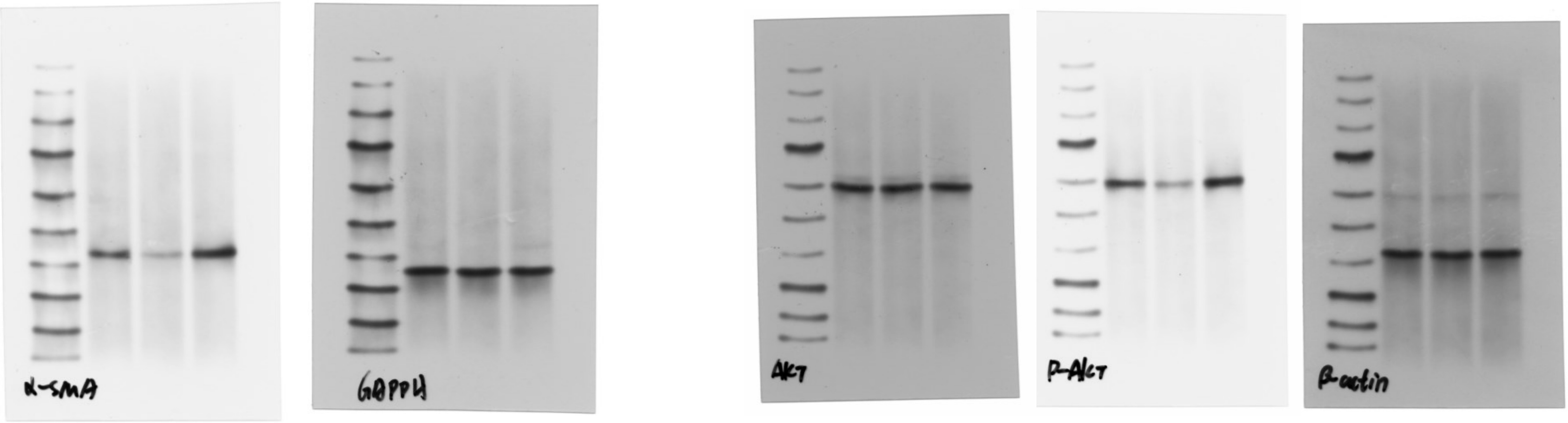

Supplement: Supplementary file 3 — Original WB images [file 41419_2025_7464_MOESM3_ESM.pdf]
